# Twelve tips for implementing artistic learning approaches in anatomy education

**DOI:** 10.15694/mep.2017.000106

**Published:** 2017-06-20

**Authors:** Iain D. Keenan, Joseph Hutchinson, Kathryn Bell

**Affiliations:** 1 Newcastle University; 2 Newcastle upon Tyne Hospitals NHS Foundation Trust

**Keywords:** Anatomy, Artistic learning methods, Undergraduate medical education

## Abstract

This article was migrated. The article was marked as recommended.

To enhance learning and maximise student satisfaction while simultaneously optimising costs and resources within the modern context and environment of integrated anatomy education, it is vital that innovative methods of delivering learning and teaching are considered for implementation into medical curricula. The development of learning processes including observation, visualisation, haptic reasoning and visuospatial ability are strongly associated with the use of artistic approaches. In addition to being crucial for medical student learning of anatomy and other life sciences, such skills are also relevant for training in surgery, clinical observation and diagnosis. The twelve tips outlined here have been identified with the intention of providing guidance for anatomy educators aiming to incorporate innovative creative and artistic approaches into their own teaching practice within medical curricula. These proposals are underpinned by educational theory and recent research that has investigated artistic learning methods in medical education. Recommendations are also based on our personal experiences from both the undergraduate student point of view as well as the academic educator perspective with respect to the usage of creative and artistic learning approaches in anatomy education at Newcastle University.

## Tip 1: Take a scholarly approach

When designing and introducing any teaching innovation, in order to provide the best possible educational experience for students, it is important to draw on many aspects of the learning process and environment. To provide a strong pedagogical basis for an artistic learning approach, it is important to identify both the educational theory and research evidence that underpin the practical method. Theories of learning can provide an effective starting point when designing an innovative artistic approach, particularly those that include scope for learning that begins with a basic outline and then gradually builds upon prior knowledge of anatomical structures in order to develop complexity ( Ausubel, 2012; Kolb, 1984), a process that can correspond to the production of artwork. Theories of drawing and learning through art ( Catterall, 2005; Perkins, 1994; Petherbridge, 2010) can also provide useful insights into the practical educational value of artistic methods. The pedagogical value of artistic methods are primarily related to the motor skills and cognitive processes that are engaged when using such approaches. Research evidence suggests that key elements of observation ( Backhouse, Fitzpatrick, Hutchinson, Thandi, & Keenan, 2017; Chamberlain et al., 2014; Jasani & Saks, 2013; Lyon, Letschka, Ainsworth, & Haq, 2013; Moore, Lowe, Lawrence, & Borchers, 2011; Pellico, Friedlaender, & Fennie, 2009) reflection and repetition ( Backhouse et al., 2017; Lyon et al., 2013), and visualisation ( Pandey & Zimitat, 2007) should be incorporated and emphasised within any artistic learning approach. Haptic ( Jones, Minogue, Tretter, Negishi, & Taylor, 2006; Woods & Newell, 2004) and visuospatial abilities ( Garg, Norman, & Sperotable, 2001; Nguyen, Mulla, Nelson, & Wilson, 2014; Nguyen, Nelson, & Wilson, 2012) should also be considered. It can also be beneficial to explore the additional benefits of artistic approaches beyond anatomy learning. For example, the observational skills that are developed through the use of artistic methods can also be transferable to clinical practice ( Bardes, Gillers, & Herman, 2001; Boudreau, Cassell, & Fuks, 2008; Jasani & Saks, 2013; Kirklin, Duncan, McBride, Hunt, & Griffin, 2007; Naghshineh et al., 2008; Pellico et al., 2009). In order to implement a novel or innovative method into your curriculum, it may be necessary to convince others of the value of your approach. It is also important to conduct pedagogical activities in a scholarly manner while seeking to understand your novel approach from the student perspective. It is therefore vital to then obtain evidence that supports the educational value of your process through rigorous research and evaluation.

## Tip 2: Choose your artistic method wisely

**Figure 1.**
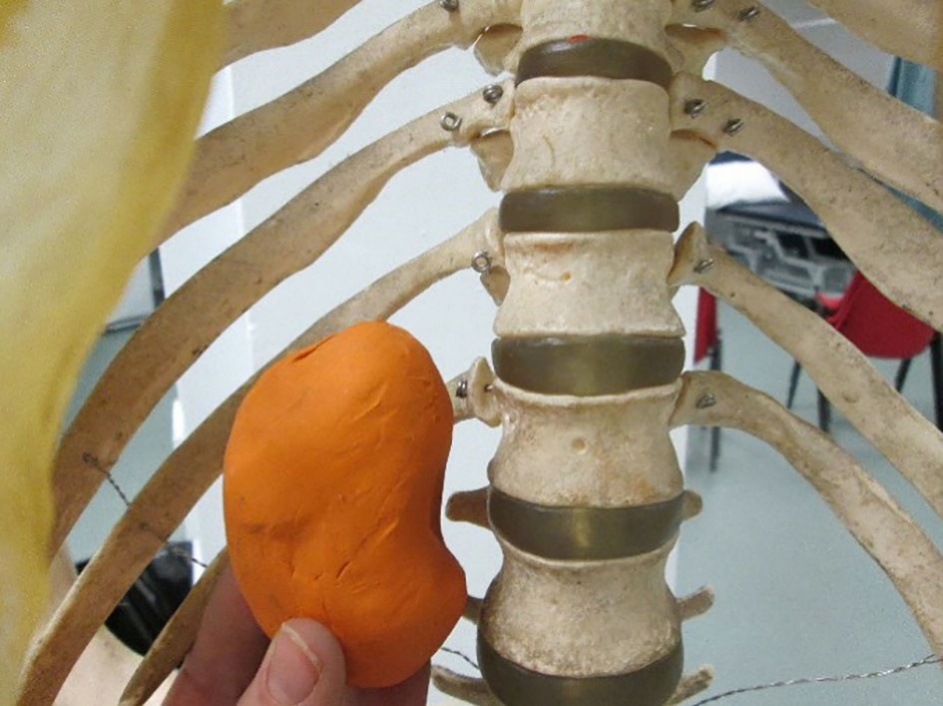
Three-dimensional models can demonstrate the size, shape and positional information of visceral organs relative to surface landmarks (Image: Amy Tiri, Newcastle University).

The specific type of artistic method used is likely to depend upon the reasons of the individual or degree programme seeking to implement such an approach into teaching, but any method should primarily be informative as well as engaging. While many artistic methods are available, it is likely that simple line drawing would be the approach utilised by most anatomy educators. This is a method already commonly used by educators and students alike in the delivery of teaching as well as self-directed learning and revision. Drawing also has the benefit of a vast history of anatomical illustration that culturally supports the popularity of this method ( Ghosh, 2015). Drawing has received relatively greater attention in terms of primary research in anatomy education that other artistic methods, with several recent studies investigating the benefits of this particular technique ( Ainsworth, Prain, & Tytler, 2011; Backhouse et al., 2017; Balemans, Kooloos, Donders, der Zee, & Catharina, 2016; Lyon et al., 2013; Naug, Colson, & Donner, 2011; Nayak & Kodimajalu, 2010). Learning through drawing has also been investigated in terms of educational neuroscience and cognitive processing ( Chamberlain et al., 2014; Guérin, Ska, & Belleville, 1999) and has certain theoretical underpinnings ( Petherbridge, 2010). The human body exists in three-dimensions, and so unlike drawing, which produces 2D outputs, modelling techniques can be valuable for creating 3D representations of anatomical structures ( Bareither et al., 2013; Cavalcanti de & Martins, 2013; DeHoff, Clark, & Meganathan, 2011; Kooloos, Schepens-Franke, Bergman, Donders, & Vorstenbosch, 2014; Motoike, O’Kane, Lenchner, & Haspel, 2009; Naug et al., 2011; Oh, Kim, & Choe, 2009; Palombi, Pihuit, & Cani, 2011). For example, a simple model can be made to represent a visceral organ and can then placed into a skeleton to demonstrate the position of the organ relative to surface landmarks ( **Figure 1**). However, both modelling and drawing can be used in combination ( Naug et al., 2011) to enhance an understanding not only of 3D anatomy, but also of 2D clinical images. For example, when considering the anatomy of the thorax, cross-sectional drawing of this region combined with modelling of the heart can provide a basis for understanding how gross anatomy relates to clinical imaging and could therefore facilitate enhanced clinical image interpretation. Simpler models can be produced by folding of paper on which diagrams have been drawn in order to create 3D anatomical structures and dynamic processes representing for example, the nerve supply to the compartments of the thigh or the formation of the neural tube. The combined use of drawing and modelling also provides a multisensory experience whereby haptic as well as visual processes are engaged. The examples presented here are not exhaustive and alternative approaches including textile arts, music, the performing arts and creative writing ( Woodward, O’Sullivan, E Holmes, & Keenan, 2017), could also be introduced as artistic learning methods. However, it is important to recognise that the level of skill and experience required to use such methods must not be prohibitive, in order to enable both students and educators to successfully utilise them. A further popular artistic approach in anatomy education is body painting, where the surface reflections of anatomical structures are painted onto the skin ( Bennett, 2014; Finn & McLachlan, 2010; McMenamin, 2008). This method has specific practicalities with respect to implementation which have been addressed extensively elsewhere ( Finn, 2010).

## Tip 3: Choose your materials wisely

**Figure 2.**
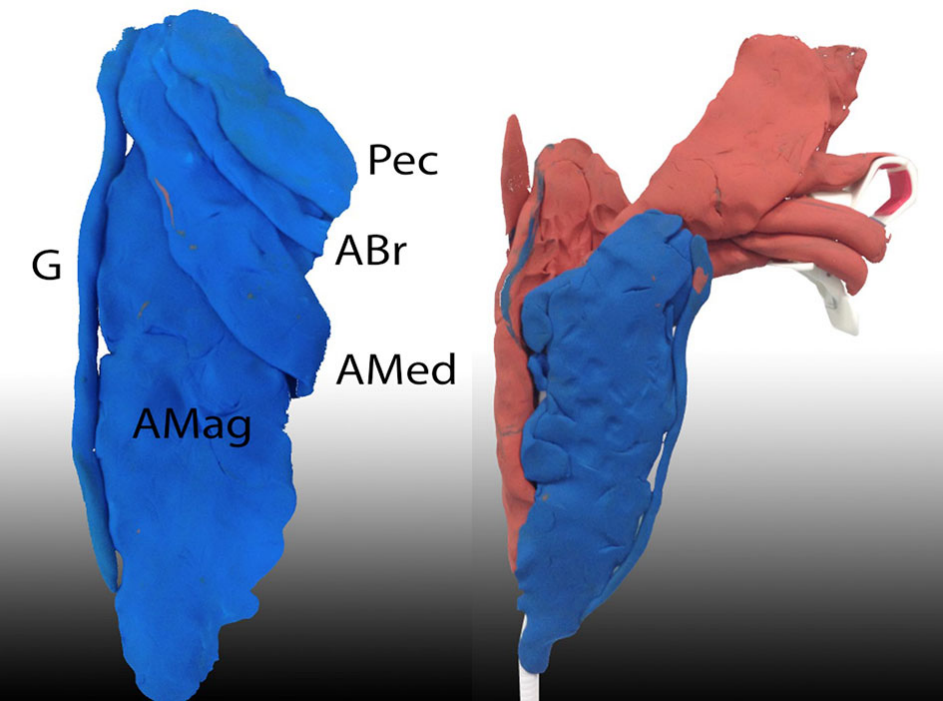
Modelling clay attached to a mobile phone stand to represent thigh muscles attached to the femur and hip joint. A Mag = Adductor magnus, A Med = Adductor medius, A Br = Adductor brevis, G = Gracilis, Pec = Pectineus. (Image: Jocelyn Selwyn-Gotha).

Artistic methods do not require the same level of staffing, materials and consumables to implement as the use of human cadaveric material ( Aziz et al., 2002; McLachlan, 2004; McLachlan & Patten, 2006). Nor are they associated with the large costs associated with the purchase of new technologies such as those required for 3D printing ( Li et al., 2017), but some additional equipment and resources will likely be required in order for the approach to be implemented successfully. The type of materials used will obviously depend upon the method used ( **Tip 2**) as well as the purpose ( **Tip 4**) of the approach. When used primarily as a learning process, pencils and paper would likely be sufficient for drawing exercises, which may be advantageous when choosing this method. As the level of creativity and freedom involved in drawing approaches increases, additional and higher quality materials are likely to be required. If modelling is chosen, the type of material used can be important to the success of the activities. In addition to sourcing the materials, pottery clay modelling requires specific facilities and equipment to support it and improvisation with commonly available materials can therfore be effective. For example, to construct a lower limb model, modelling clay can be attached to a mobile phone stand as a bone template ( **Figure 2**). Some commercially available modelling clays are nominally reusable, but can be challenging to manipulate when used at room temperature, can easily become contaminated when used alongside cadaveric material and colour mixing can be an issue. Less expensive and disposable modelling materials can be made in-house with flour, salt, water and food colouring. An association between the use of colour and impact on learning has been identified ( Dzulkifli & Mustafar, 2013; Finn, White, & Abdelbagi, 2011), so the use of coloured materials in the context of whichever method is chosen is recommended. Depending on the method, it is important to consider that a location outside of the dissecting room suitable for use as a studio or theatre, and a certain level of external expertise ( **Tip 9**) may be required to successfully implement the approach.

## Tip 4: Ensure a clear purpose

**Figure 3.**
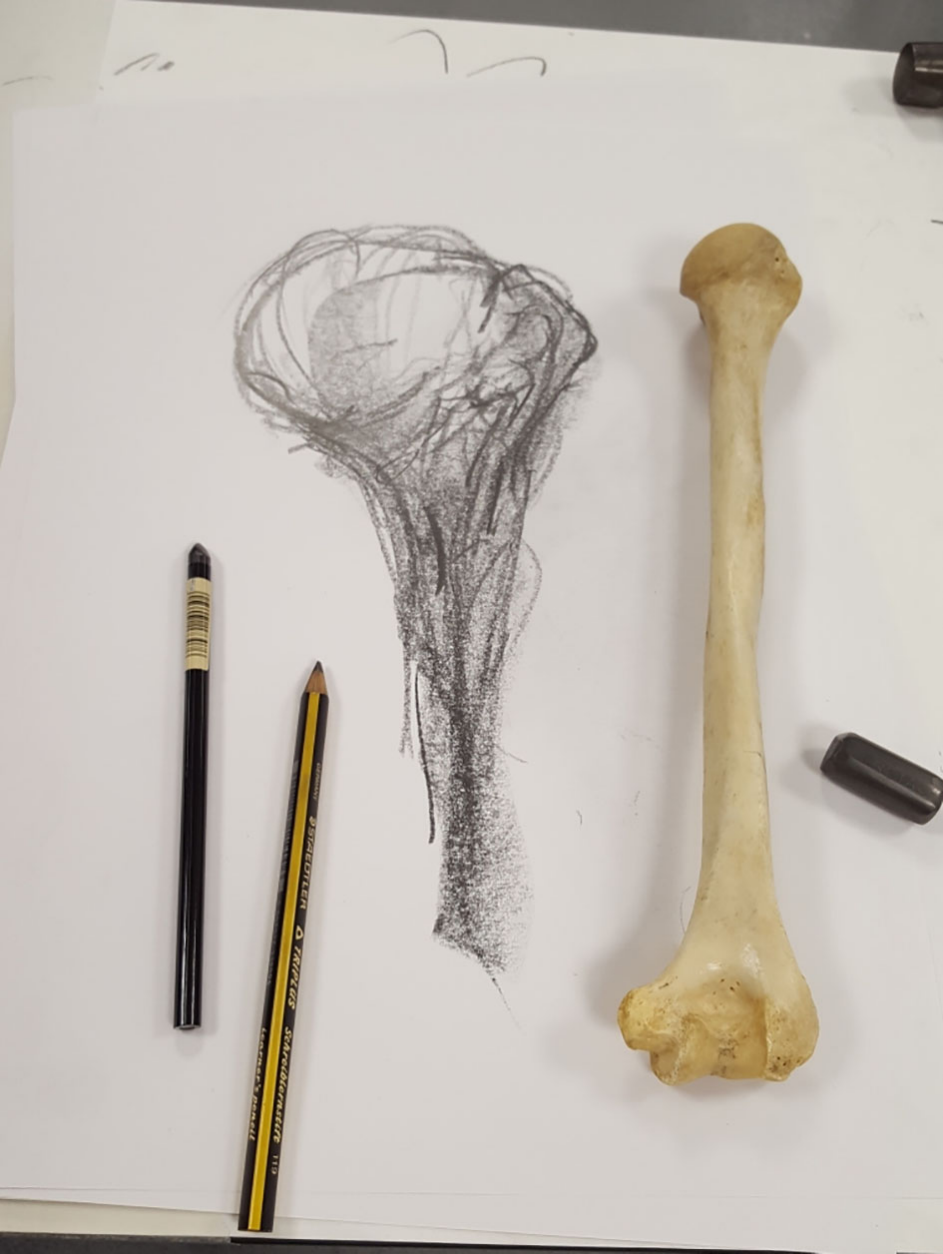
*Artatomy* exhibition 2015 at Science at Life Centre, Newcastle upon Tyne. (Image: Matthew Horne, Newcastle University).

Whichever artistic method is chosen and used ( **Tip 2**), it is important to understand the purpose of using it from the outset. Has the approach be chosen as **A**) a specific tool to facilitate the learning process or **B**) for broader creative and holistic purposes? Addressing this question with clarity is required from both the student and educator perspective. Both approaches **A**) and **B**) can be valuable for students but it is important to explicitly ensure that only one approach is used in any one teaching session or situation and to explicitly inform students which method is being used at that time, so that the purpose of the activity is clear. It is also crucial to define whether the chosen approach is provided as an optional opportunity for those medical students who are interested or whether it is intended for integration into formal teaching for the entire cohort. For example, there is a clear difference between our *Observe-Reflect-Draw-Edit-Repeat* (ORDER) artistic learning process ( Backhouse et al., 2017), and our creative *Artatomy* project ( **Figure 3**). *Artatomy* has been effective for providing medical students with an opportunity to view human anatomy and therefore their patients from alternative viewpoints, which we have achieved through the implementation of optional drawing workshops and art exhibitions. In our experience, medical students are increasingly focused on examination scores at the expense of other valuable pursuits that can contribute to the development of well-rounded clinicians. *Artatomy* has provided scope for students to consider anatomy out with the context of their studies and therefore unrelated to assessment. This approach is distinct from ORDER, which we designed as a formal learning process for the acquisition of knowledge with respect to specific anatomical structures and concepts in anatomy practical sessions.

## Tip 5: Emphasise learning gain over aesthetic creation

Students will only experience the full value of enhanced skills and knowledge when using artistic methods if they are encouraged to participate in artistic activities. Rather than providing artistic methods purely as a didactic demonstration, it is therefore important to encourage active learning in order for students to connect with the discipline and for learning gain and engagement to be optimised (Freeman et al., 2014; Hake, 1998). Furthermore, it is important to design practical activities that students participate in themselves, whether that be active observation, visualisation using drawing, modelling or any combination of processes and methods. However, it is important that students need not to be talented in the artistic method and must also not be intimidated by it. In the context of implementing purpose **A**) ( **Tip 4**), when faced with a drawing task in a formal teaching session, most students will claim they are not artistic or cannot draw ( Backhouse et al., 2017). This is perhaps the most challenging aspect of introducing artistic learning methods into curricula, but fortunately it can be addressed. They key here is to emphasise to students that they are undertaking a process of learning rather than producing artwork, which some, if not all students will embrace when they realise that they do not need to be “artistic” in order to learn in this way. It can even be valuable to refrain from using terms such as “art”, “artistic” or “artwork” when describing purpose **A**) learning activities to students. Those students who enjoy artistic activities may be distracted by their attempts to produce an aesthetically pleasing image, so it is equally important to address the learning process of these particular students. Depending on the task, it can sometimes be helpful to suggest that students immediately throw away their drawings having completed them in order to emphasise process over product. As with any innovative learning activity, not all students will be convinced of the value of artistic learning methods. It is therefore important to either embrace their viewpoint, which can be achieved through providing alternative “non-artistic” strategies, or perhaps even by motivating student engagement through assessment.

## Tip 6: Know your audience

It is important to recognise the differences between individual students as well as their similarities. Within the specific context of artistic approaches, student confidence in their artistic abilities is likely to directly impact upon their engagement in the approach. In addition to emphasising learning gain ( **Tip 5**), introducing artistic methods gradually over an extended period, perhaps beginning with a brief explanation of the purpose followed by a short warm up activity involving a simple line drawing ( Lyon et al., 2013) as part of a wider session is likely to increase confidence and engagement. Developing and building upon this approach throughout the curriculum would then likely to be valuable and appreciated by students. In terms of the student population, we have shown that artistic methods are not only effective for the learning of those students who are “artistic” ( Backhouse et al., 2017). However, if students do not consider themselves to be “artistic”, they may still believe that they cannot learn in this way, even if learning is emphasised over artistic creation in the context of purpose **A**). It is therefore important to explicitly state to students that this is not the case. We have also shown that the ability to learn using artistic methods is independent of student preferences for learning ( Backhouse et al., 2017). However, while the notion of learning styles has been effectively discredited (Coffield, 2004; Hall, 2016; Rohrer & Pashler, 2012), we identified that students will assume that a “visual learning style” is required for their successful utilisation of artistic methods when studying anatomy. Again, it is important to inform students that this is not the case, and emphasise that leaving their own comfort zone can actually be effective for their learning. Actively encouraging reluctant students to engage in the artistic activity may help them to realise that they can actually learn in different ways ( Naug et al., 2011), which is important for their metacognition and lifelong learning beyond anatomy and medical school. While artistic background and learning preferences may not influence learning with artistic methods, it is important to be aware of the existence of gender differences in visuospatial ability ( Langlois et al., 2013; Linn & Petersen, 1985), an attribute which in turn is a key indicator of anatomical understanding ( Fernandez, Dror, & Smith, 2011; Guillot, Champely, Batier, Thiriet, & Collet, 2007; Rochford, 1985; Vorstenbosch et al., 2013).

## Tip 7: Provide clear guidance

As with any innovative method, students require sufficient information in order to be successful when practically utilising novel approaches for learning. It is also important to ensure that the delivery of core anatomy content ( Smith et al., 2016) is not sacrificed at the expense of the method itself. It may seem obvious, but it is important to state that students will still require teaching alongside the use of artistic methods so that they are able to understand the necessary anatomical structures and concepts as well as learning how to use a new method. This can be achieved, for example, by ensuring that drawings are labelled accurately and that structures are identified on models as required. While particular skills are addressed and enhanced through the use of artistic methods ( **Tip 1**), explicitly providing explanations of the meaning of terms and active usage of processes such as observation, reflection and visualisation to students is essential if they are to fully appreciate the purpose of the activity and are able to perform each task correctly, and are therefore able to optimise their learning by doing so. For example, it is important to explain that the term “observation” means more than simply looking at an anatomical specimen. If this is not understood then then the educational value of observation may not be appreciated by students. Observation can be haptic as well as visual and requires that the student pays close and critical scrutiny in terms of the 3D form and structure of the anatomy over and above the initial superficial appearance, in order to allow them to understand and retain the information ( Backhouse et al., 2017; Lyon et al., 2013). An ordered and clearly defined protocol for carrying out the activity can also emphasise learning over artistic creation ( **Tip 5**), when purpose **A**) is intended. As the educator, it is important to be able to deeply understand the practical aspects of the artistic method and to be able to demonstrate an accurate and informative example of how it should be used. This is best achieved through repeated use and practice prior to implementation. From the student perspective, it is not essential that the educator is “artistic” or “able to draw”, only that the teacher is able to successfully demonstrate the method. In some cases, being “non-artistic” may actually serve the educator well as an example to students of the limited extent of the artistic talent required to complete the task and to successfully learn from it.

## Tip 8: Cultivate student partnerships

In addition to engaging in learning and participating in research, student partners ( Healey, Flint, & Harrington, 2014) can provide effective insights from their own perspective through offering creativity and diligence in terms of the design, evaluation and implementation of artistic learning methods in anatomy education. While the general value of peer-peer and near peer learning in anatomy has been described ( Border et al., 2017), recent studies have successfully included undergraduate students in key aspects of the implementation and evaluation of artistic learning approaches ( Backhouse et al., 2017; Lyon et al., 2013; Naug et al., 2011; Nayak & Kodimajalu, 2010). Such partnership strategies can also provide students with the opportunity to acquire skills and knowledge in medical education research and teaching, as well as contributing to their personal and transferable skill development that will serve them well in their future clinical careers. The opportunity to deliver teaching using methods they have designed and to present their research findings at conferences are undoubtedly important experiences in their development. The establishment of undergraduate student partnerships can be achieved during summer projects, by working with student societies or through working informally with students on an ad hoc basis. Running related projects simultaneously can create a small community of effective collaboration for student partners. While projects can be brief, committed students who have acquired intellectual and emotional investment through actively and productively contributing to partnerships are often motivated to pursue further projects both during and beyond their medical studies.

## Tip 9: Cultivate artist partnerships

**Figure 4.**
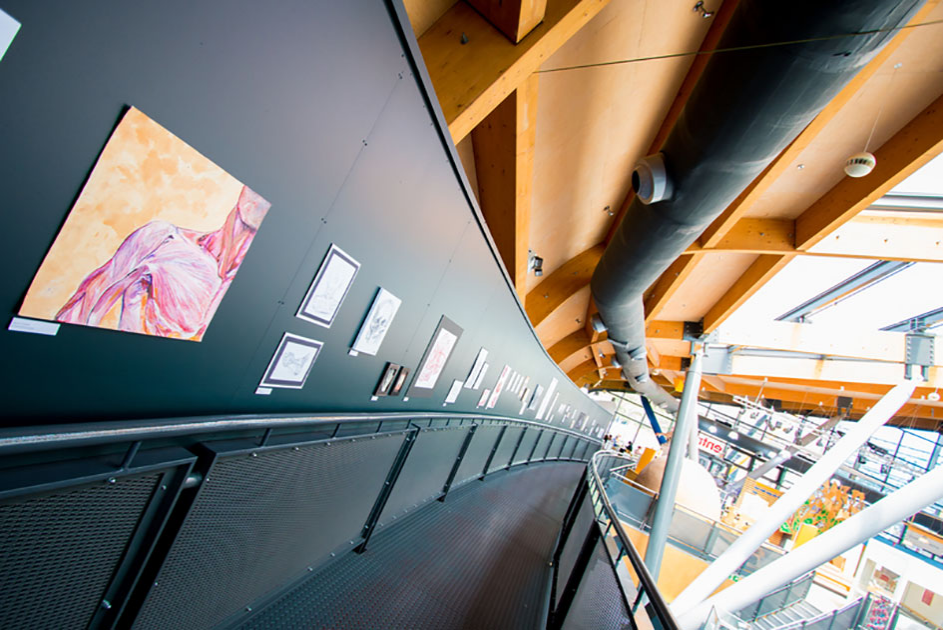
Drawing of a humerus from a haptico-visual observation and drawing workshop facilated by professional drawing instructor Leonard Shapiro in May 2017 (Image: Iain Keenan).

While student demonstrators can enhance learning of their medical student peers, art students can also provide support to participating students. However, it is important to ensure that like student participants, art students are given appropriate guidance and do not present a conflict with the process over product message ( Backhouse et al., 2017). For this reason, art student partners are likely to be most effective in purpose **B**) approaches. In addition to providing creative input into the theoretical basis, design and subsequent implementation of artistic methods in purpose **A**) approaches, professional artists and drawing instructors are able to demonstrate the artistic process and how to successfully utilise it in purpose **B**) activities. Such practical demonstrations may also be used with purpose **A**), for example in warm up drawing exercises and the use of appropriate instruments, materials and resources. Experienced artist contributors can also facilitate the understanding of learning processes of visual and haptic observation and visualisation and can train both academics and students in these processeses ( **Figure 4**). With respect to **Tip 4** and **Tip 5**, it is crucial that the professional artist involved understands the purpose of the approach, so as not to communicate conflicting messages to student participants.

## Tip 10: Use artistic methods to supplement and provide variety

It is important to provide a variety of activities in order for learning to be optimised ( Eagleton, 2015; Sugand, Abrahams, & Khurana, 2010; Ward & Walker, 2008) and artistic methods can be implemented as one of several approaches with a view to providing this variation. It is unlikely that anatomy educators would rely heavily on artistic methods to deliver the majority of their teaching and it is recommended that this should continue to be the case. Rather, artistic methods should be used to complement and supplement learning rather than as an attempt to replace existing methods, as a variety of traditional approaches will provide greater educational value than a single artistic method ( Backhouse et al., 2017). This may be organised as an individual artistic anatomy session in the context of a wider curriculum, or preferably, could be designed whereby artistic methods are utilised as a single component across multiple anatomy sessions. Depending on the intended purpose, **A**) or **B**), self-directed resources or optional workshops can be effective for the delivery of anatomy using artistic methods, while supplementary activities within practicals or even lectures can provide welcome alternatives to traditional teaching when scheduled at a time when engagement and concentration are waning. Students feedback certainly indicates that students prefer a supplementary approach to artistic methods over extended and intensive delivery ( Backhouse et al., 2017).

## Tip 11: Optimise timing

While providing clear guidance and framework for the artistic method is important ( **Tip 7**), it is also possible to deliver the approach in a flexible manner that does not require students to adhere too strictly to instructions and timings. However, sufficient time on task is essential for optimal achievement in any learning activity, including those in anatomy ( Chickering & Gamson, 1987; Fasel, Morel, & Gailloud, 2005; Gibbs, 2010). Artistic methods in particular require an appropriate amount of time to complete the activity, whether that be the drawing, model or other creation. It is also important to allow sufficient time for students to produce drawings collaboratively ( Backhouse et al., 2017; Bergman et al., 2013; Lyon et al., 2013) and while team-based activities can be very effective, they can time consuming to implement during a session. However, putting strict time limits on activities can restrict learning. For example, if students have only reached the stage where they have drawn a diagram but have not yet had time to discuss it or label it then the extent of learning and value of the activity is also restricted, and this recognised by students ( Backhouse et al., 2017). Sufficient time spent on the specific learning processes including observation, visualisation and reflection are also required.

## Tip 12: Incorporate technology

When considering **Tip 10** and **Tip 11** together, one solution to providing flexible and supplementary approaches to learning using artistic methods can be achieved through utilising interactive online tutorials. Such resources can allow students to progress at their own rate through the process without the pressures of time and also remove the requirement for including many artistic methods within curricular teaching sessions. We found that delivering our ORDER process in online videos also resulted in enhanced learning using this particular artistic method, when compared to our findings in the context of anatomy practical sessions ( Backhouse et al., 2017). Our interactive tutorial also provided an opportunity to include formative assessment through multiple choice questions, in addition to testing and questionnaire functionality which allowed us to efficiently evaluate learning and student perceptions. Social media have been successfully utilised for anatomy learning and as a supplement to medical education ( Bahner et al., 2012; Hennessy, Kirkpatrick, Smith, & Border, 2016). These resources provide an accessible platform where the educator can faciliate the creation of a community for those students who choose to use artistic learning methods. This can be achieved through the lecturer posting images of their own drawings, models or other products of the artistic process which display anatomical accuracy emphasised over aesthetic appearance (Tip 5), as learning resources for purpose **A**), while social media can also be used to and exhibit artwork in the context of purpose **B)**. For example, student competitions where they are asked to design and post their own creative learning methods can be particularly effective when facilated by social media and can result in increased engagement during the delivery of challenging topics such as embryology.

## Take Home Messages

## Notes On Contributors

Dr Iain Keenan is a Lecturer in Anatomy based in the Anatomy and Clinical Skils Centre within the School of Medical Education at Newcastle Univeristy. In addtion to delivering teaching for undergraduate and postgraduate medical, medical sciences and medical education degree programmes, his research focus concerns the design, development and evaluation of innovative creative, artistic and digital approaches to anatomy learning.

Dr Joseph Hutchinson completed his medical training at Newcastle University, UK, graduating in Summer 2016. During his undergraduate years he was afforded an undergraduate research scholarship to study novel applications for the ORDER technique of learning. This focussed on using e-learning as a platform for revision tutorial delivery; with narrated videos to help guide students through a plasticine modelling process. He also contributed to this work during two further undergraduate projects and was involved in the development and implementation of *Artatomy*, a student-led exhibition of anatomical artwork. He is now working as a foundation doctor in the Newcastle-upon-Tyne NHS Foundation Trust, Hepato-Pancreato-Biliary Centre. His future academic interests include the further use of electronic technology in medical education and healthcare delivery, concentrating on a national and international context.

Kathryn Bell is a fourth year undergraduate medical student at Newcastle University with an interest in anatomy and medical education. She pursued an undergraduate project in the School of Medical Education in 2017 and contributed to the design and development of research surrounding a haptico-visual obervation and drawing workshop held at Newcastle University in May 2017.

## Declarations

Dr Iain Keenan declares a conflict of interest as current theme guest co-editor of “Life Sciences in an Integrated Curriculum” in MedEdPublish. There are no other conflicts of interest to declare.
