# Supplementary material for: Twelve tips for implementing artistic learning approaches in anatomy education
Source: MedEdPublish (2016). 2017 Jun 20;6:106. [Version 1] doi: 10.15694/mep.2017.000106 (PMC10885264; doi:10.15694/mep.2017.000106)
Supplement: Peer review history

# Reviewer report: Reviewer response for version 1

**DOI:** 10.21956/mep.19254.r27961

**Published:** 2019-03-07

**Authors:** Trevor Gibbs

**Affiliations:** 1 AMEE

This review has been migrated. The reviewer awarded 5 stars out of 5 I think that anyone working on developing an undergraduate medical or health professions curriculum knows the importance of a solid grounding in anatomy. Our problems come from deciding on the depth of learning and the mode of teaching. This clearly written paper tackles the latter point and shows how to introduce more active learning into the subject. I enjoyed reading the paper and learned about some new, novel methods. I also valued the list of references regarding this topic, very comprehensive.I would be very happy to recommend this paper to all those involved in anatomy teaching and curriculum development.I would be v

Reviewer Expertise:

NA

No decision status is available

# Reviewer report: Reviewer response for version 1

**DOI:** 10.21956/mep.19254.r27960

**Published:** 2019-01-26

**Authors:** Ken Masters

**Affiliations:** 1 Sultan Qaboos University

This review has been migrated. The reviewer awarded 4 stars out of 5 A well written paper indeed. The authors skilfully blend educational, anatomical and artistic theory and practice into an easy-readable and useful guide. Their suggestions are firmly grounded in both literature and their own experience, and nicely supported by their illustations. Well done.A criticism on the layout: Each tip consists of a wall of text. I would like to see the authors break the walls into smaller paragraphs in order to ease the reading.

Reviewer Expertise:

NA

No decision status is available

# Reviewer report: Reviewer response for version 1

**DOI:** 10.21956/mep.19254.r27962

**Published:** 2017-06-21

**Authors:** PATRICIA CURY

**Affiliations:** 1 FACERES

This review has been migrated. The reviewer awarded 4 stars out of 5 This is a very interesting paper with many tips to make basic science, like anatomy, more interesting to the students. We estimulate our students in basic patholgy to use criativity in their seminars, trying to transform the subject such as "acute inflamation" in a clínical case of acute apendicitis. They perform videos, music and theatre to explain the theme to other students. All enjoy them, and emotion fix more the information for them, as everybody knows.

Reviewer Expertise:

NA

No decision status is available
